# EHV-1 Pathogenesis: Current *in vitro* Models and Future Perspectives

**DOI:** 10.3389/fvets.2019.00251

**Published:** 2019-07-31

**Authors:** Mohamed Kamel, Selvaraj Pavulraj, Klaus Osterrieder, Walid Azab

**Affiliations:** ^1^Institut für Virologie, Zentrum für Infektionsmedizin, Freie Universität Berlin, Berlin, Germany; ^2^Department of Medicine and Infectious Diseases, Faculty of Veterinary Medicine, Cairo University, Giza, Egypt

**Keywords:** equine herpesvirus, pathogenesis, *in vitro*, model, EREC, flow chamber, *ex vivo* explant

## Abstract

Primary infection and pathogenesis of equine herpesvirus type 1 (EHV-1) require an intricate interaction of virus with the mucosal epithelium, mononuclear cells and the vascular endothelium. Studies on EHV-1 have been facilitated by the development of different *in vitro* models that recapitulate the *in vivo* tissue complexity. The available *in vitro* assays can be categorized into (i) models mimicking the epithelium-peripheral blood mononuclear cell (PBMC) interaction, which include *ex vivo* mucosal (nasal and vaginal) explants and equine respiratory epithelial cells (EREC) cultures; and (ii) PBMC-endothelium mimicking models, including flow chamber and contact assays. These *in vitro* models have proven their worth in attempts to recapitulate the *in vivo* architecture and complexity, produce data relevant to natural host infection, and reduce animal use due to *in vivo* experiments. Although horse models are still needed for certain experiments, e.g., EHV-1 myeloencephalopathy or vaccination studies, available *in vitro* models can be used to obtain highly valuable data on virus-host tissue interactions. Microfluidic based 3D culture system (e.g., horse-on-a-chip) could be a potential upgraded version of these *in vitro* models for future research.

## Introduction

Alphaherpesviruses are a heterogeneous group of morphologically similar DNA viruses that includes important pathogen of humans and animals. Equine herpesviruses infect mainly members of family *Equidae*, but also members of other taxa, and cause substantial economic losses (1, 2). Equine herpesviruses 1 and 4 (EHV-1 and EHV-4) are endemic in domestic horse populations worldwide and cause respiratory conditions. EHV-1 is the prime cause of abortion, neonatal mortality and neurological disorders (myeloencephalopathy) after a transition stage of cell-associated viremia. EHV-1 infection of peripheral blood mononuclear cells (PBMC) plays an essential role in transmitting the virus from the primary site of infection (respiratory tract) or reactivation to the vasculature of target organs (3, 4).

EHV-1 pathogenesis can be divided into three main levels of infection. First, in the respiratory epithelium, the infection starts with uptake of infectious particles and primary replication in respiratory epithelia. Second, in mononuclear cells and dendritic cells (DC), virus is captured from the primary site of replication (respiratory epithelia), and infected cells rapidly migrate to lymphoid tissues associated with the upper respiratory tract and infect other mononuclear cells that enter the blood stream (cell-associated viremia). Finally, the virus is transferred from blood mononuclear cells to the vasculature of different tissues, where viruses can attach to, enter and replicate in endothelial cells (EC). Disease outcomes are reflecting the pathogenetic changes including vasculitis, thrombosis, edema and vascular necrosis (4–10). The process of virus spread between the three compartments (epithelium, blood, and endothelium) is dynamic and involves multiple steps and it is critical that the orchestration of these steps be precisely regulated to ensure efficient virus transfer. However, the exact mechanism at each level is still unknown and needs further investigation.

Of course, horses are the gold standard for studying EHV-1 pathogenesis; however, this model is ethically questionable and requires large animal biosafety facilities and trained personnel. Absence of other suitable animal models resulted in the development of *in vitro* systems to study EHV-1 pathogenesis. The currently available *in vitro* models can be divided into two main categories. (i) Epithelium-PBMC mimicking models, which include *ex vivo* nasal explants and equine respiratory epithelial cells (EREC) culture; (ii) PBMC-EC mimicking models, which include flow chamber assay and contact assay. These models are widely used to recapitulate *in vivo* architecture and investigate the host-pathogen interaction.

## *Ex vivo* Nasal Explants

Nasal explants provide an attractive and alternative means to mimic the *in vivo* situation as a complex 3-dimentional tissue network that keeps intact the cell-to-cell contacts present *in vivo*. This model is readily accessible and is a powerful tool to overcome problems when using infection experiments in the natural host, including, but not restricted to, better standardization and the possibility to perform multiple replicates that are impossible in the horse infection model (11). Nasal explant cultures have been successfully used to study the pathogenesis, replication and invasion of EHV-1, EHV-3 and EHV-4, the initial response and migration of mononuclear cells during EHV-1 infection, basement membrane damage during infection, and EHV-induced cytokine responses (7, 12–16). Confocal microscopy studies showed that EHV-1 crosses the basement membrane barrier through infected mononuclear cells, which allows the virus to subsequently progress to draining lymph nodes or blood vessels in lamina propria and results in cell-associated viremia (7, 14). Migration of mononuclear cells in response to virus infection or navigation of infected cells to blood vessels or lymph nodes is determined by complex network of cellular signals and the actions of cytokines and chemokines.

Vaginal mucosal explants are a variant version of mucosal explant cultures that were used to study EHV-1 and EHV-3 entry, replication kinetics, virus spread and invasion characteristics. Although both viruses can replicate efficiently, EHV-3 showed privileged replication in the vaginal compared to nasal mucosa due to natural virus tissue tropism (17).

## EREC Culture

**Figure 1:**
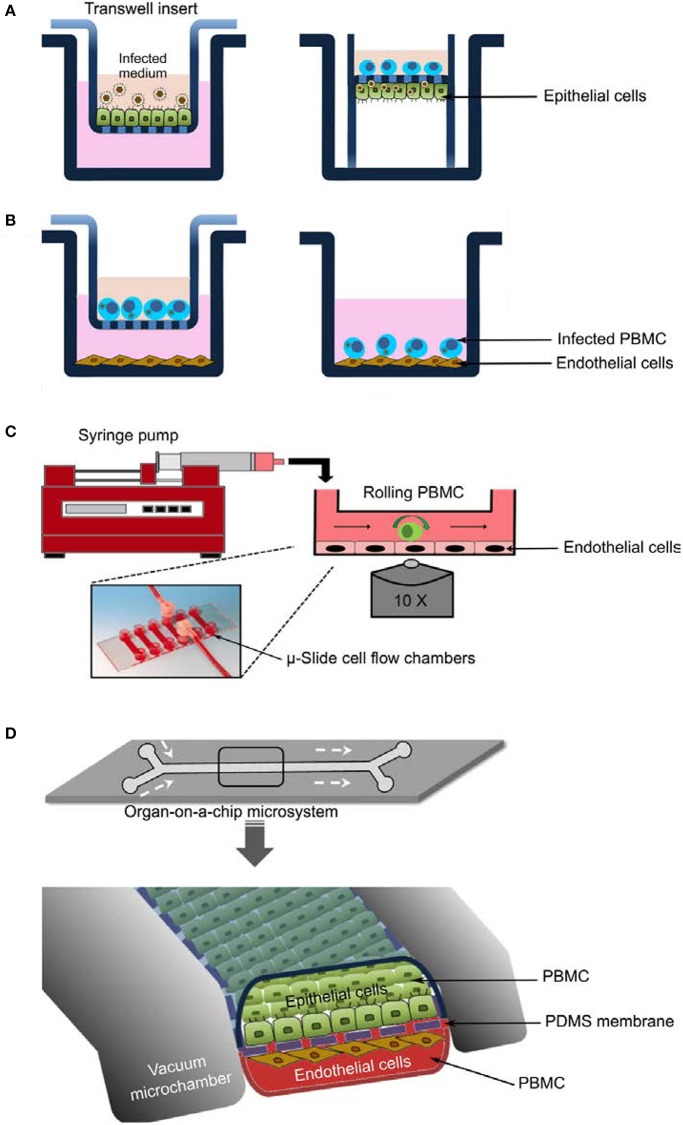
*In vitro* models to study EHV-1 pathogenesis. **(A)** Equine respiratory epithelial cells (EREC) culture. Cells are grown at the air-fluid interface and infected with virus at the apical side (left panel). After removal of the inoculum, the transwell insert is inverted, a tygon pipe is applied, and PBMC are added to the generated top chamber (right panel). **(B)** Contact assay. Virus-infected PBMC are applied to endothelial monolayers either in a “transwell; left panel” or “contact; right panel” setup. **(C)** Flow chamber system. Endothelial cells are grown to confluency in μ-slide cell flow chambers connected to a perfusion system “syringe pump” that allow the introduction of infected PBMC. PBMC kinetics as well as PBMC-endothelial cell interactions can be visualized with inverted fully motorized fluorescence microscope. **(D)** Organ-on-a-chip microdevice. The microsystem is constructed in a layered microfluidic device with two cell culture (upper and lower) microchannels separated by a porous flexible membrane. Epithelial and endothelial cells are grown on the upper and lower microchannels, respectively. Growth medium, virus, or virus-infected PBMC are perfused using a syringe pump. Virus infection and transfer, kinetics of perfused PBMC, and interaction between PBMC and cell monolayers can be visualized and tracked using live cell imaging.

Polarized epithelial cells differentially distribute proteins and lipids in the plasma membrane creating two distinct surfaces: the apical surface, which faces the external environment, and the basolateral side, which contacts the underlying cells and systemic vasculature (18). Most studies on virus entry have been conducted with non-polarized cells do not properly reflect *in vivo* conditions. Epithelial cells grown on porous supports show evidence of increased differentiation in comparison with cells grown on conventional solid surfaces, which formed the base for EREC *in vitro* culture model (19, 20) and was recently adopted to study EHV-1 pathogenesis (Figure 1A) (21–23). EREC were used to study replication kinetics and cytokine response after infection with wild-type or mutant EHV-1 strains. Further, an EREC-PBMC virus transfer system was developed and has provided evidence for direct viral transfer from the epithelium to PBMC. Viral transfer through direct cell-to-cell contact resulted in pro-inflammatory, chemokine and antiviral responses that were strikingly different if each cell type was infected independently (22). In addition, the EREC system was successfully employed to shed light on chemotaxis of monocytes and neutrophils in response to EHV-1 infection of respiratory epithelial cells (8). This unique primary equine epithelial cell system closely mimics *in vivo* conditions of primary infection. Further, most of the data related to EHV-1 obtained from this *in vitro* system mimics *in vivo* data (24–26). In conclusion, the *ex vivo* models confirmed the importance of studying the cells representing different compartments of the body during infection with EHV-1 in relation to each other, rather than individually.

## Contact Assays

To mimic the PBMC-EC interface and to investigate the multitude of interactions between PBMC and EC with subsequent virus transfer, an *in vitro* co-cultivation system was developed (6). The system involved either “contact” or “non-contact” setups where both PBMC and EC are sharing the same environment in the presence or absence of neutralizing antibodies (Figure 1B). In the contact model and under static conditions, EHV-1-infected PBMC were co-cultured with EC monolayers in the presence of neutralizing antibodies, and virus transfer from PBMC to EC was reported. In the “non-contact” model, infected PBMC were placed into a transwell insert and were physically separated from EC monolayers by a porous membrane that prevents the migration of PBMC but allows the diffusion of cell-free virus (6, 9). Virus spread from infected PBMC to the underlying EC in the “contact” mode was reported and tracked using confocal microscopy and live cell imaging (9). The system proved to be flexible to study other aspects during virus spread, particularly the role of adhesion molecules in virus transmission (27).

## Flow Chamber System

To further address the more dynamic aspects of PBMC-EC interaction, we established a flow chamber setup, where infected PBMC are allowed to flow (0.5 mm/s) over EC monolayers in the presence or absence of virus-neutralizing antibodies (Figure 1C). The whole process can be tracked by confocal live cell fluorescence imaging and automated cell tracking. This system can be used to document the differences between neuropathogenic and non-neuropathogenic EHV-1 strains as well as between EHV-1 and EHV-4 (9). The role of different viral proteins in the process of virus spread from PBMC to EC was precisely addressed. The system also allowed to document the kinetics of infected vs. non-infected PBMC in terms of tethering, adhesion, and rolling. These experiments demonstrated the value of the flow chamber system for studying the dynamic events during EHV-1 transfer from infected PBMC to EC, the role of adhesion molecules, and the effects of anti-inflammatory and anti-viral treatment on virus transmission.

Another aspect of EHV-1 pathogenesis targeting the role of EHV-1 in thrombus formation was studied using another flow microfluidic system. With the system, it was possible to investigate the interaction between EHV-1 infected EC cells and platelets. The process of capturing un-activated platelets by infected EC and initiation of platelet aggregation was tracked in a dynamic mode (Tracy Stokol, personal communication).

## Conclusion and Future Perspectives

The development of *in vitro* models has paved the way to fill in the gaps in our understanding of EHV-1 and EHV-4 pathogenesis. Conducting *in vivo* experiments on horses is ethically questionable and associated with high costs that are caused by the need for specialized facilities and highly trained personnel. Furthermore, suitable replicates of experiments present a formidable hurdle. Available *in vitro* experimental models have allowed important insight into virus pathogenesis, virus-cell interactions, the crosstalk between cells, and the viral and cellular determinants governing infection.

From an animal welfare perspective, the currently available models are important steps toward reducing the suffering of horses during animal experiments, although some experiments, primarily those for vaccine development, will still require horse studies. At a technological level, the systems provide the required level of tissue complexity that is needed for a better understanding of virus pathogenesis; however, upgrades are still required to mimic and recapitulate the complicated *in vivo* situation.

The development of the horse-on-a-chip would be the suitable upgrade and may represent the future of pathogenesis models. The model would depend on the fabrication of two parallel microchannels with a thin, porous, and flexible polydimethylsiloxane (PDMS) membrane to recreate tissue-tissue interfaces. Epithelial cells and microvascular endothelial cells would be grown on the upper and lower microchannels on the collagen-coated PDMS membrane (Figure 1D). Different models of specialized tissues (e.g., blood vessels, gut, liver, kidney, lung, and brain) can now be commercially microfabricated. To study virus infection and transfer, infected PBMC would be allowed to flow with the medium over the epithelial or endothelial monolayers in the presence or absence of neutralizing antibodies. PBMC interaction with both monolayers could then be visualized and tracked using live cell imaging as described above. The flow kinetics of infected PBMC as well as virus transfer can be also studied (28–30).

Mesenchymal stem cells (MSC) are an attractive model that have been explored recently to study the pathogenesis of EHV-1. MSC have the potential to differentiate into any type of cells of mesodermal origin (31). Equine MSC can be derived from variety of sources such as amniotic fluid, umbilical cord blood, peripheral blood, bone marrow, adipose tissue, or gingival and periodontal ligament (32, 33). It was shown that MSC cells are permissive for EHV-1 lytic infection and that complete virus replication cycles can take place in these cells (34). The Self-renewable and multi-potent capacities of MSC (35) with their potential to be used in 3D cell culture/organoid platforms make them a useful tool to further study *in vitro* organogenesis and disease modeling. Given the fact that clinical disorders associated with EHV-1 infection are due to ischemic tissue injury (36, 37) and that MSC can promote angiogenesis (34), MSC based-organoids may provide an advanced *in vitro* tool that enables more physiologically-relevant experiments to be performed.

Three (3D) cell culture matrices are now widely accepted as highly complex and dynamic systems that promote many biological relevant functions through properly regulated cell-cell and cell-matrix interactions, and the dynamic distribution of oxygen, nutrients and other molecules. Currently, there are several reports that have confirmed significant differences in the morphology, viability, response to stimuli, gene and protein expression, proliferation, migration, and functionality of cells between 3D and 2D cell cultures (Table 1), which support the transition from 2D to 3D cell culture systems (38–40). Microfluidic technology can create a controllable, reproducible and optimizable dynamic microenvironment that mimics the *in vivo* environment and provides efficient and high throughput cellular analysis and *in situ* monitoring of cellular events (Table 2). The combination of microfluidic technology with 3D cell culture has great potential for *in vivo*-like tissue-based applications. This system has been widely used to study cell biology for biomedical applications, genetic assays, protein studies, intracellular signaling, multidrug resistance, drug toxicity, inflammatory responses, early-response cytokines, activation of vascular endothelium, up-regulation of adhesion molecules, and pathogen detection (29, 30, 38, 40, 41). A well-designed horse-on-a-chip microdevice could combine microfluidics and biotechnology techniques, represent alternatives to mimic the multicellular architectures, tissue-tissue interfaces, and physicochemical microenvironments. Such system will provide better levels of tissue and organ functionality compared with conventional cell culture systems, and have great potential to advance the study of disease etiology and drug discovery and development.

**Table 1:** Main differences between 3D and 2D culture systems.

	**Three dimensions (3D) cell culture**	**Two dimensions (2D) cell culture**
Merits	Closely mimic the *in vivo* microenvironment; particularly, cell-cell and cell-extracellular matrix interactions, communication, and signaling pathway	Cells are grown in monolayers, which allow them to receive equal amount of nutrients and growth factors
	Multicellular system: it provides an opportunity to co-culture multiple cell types to mimic the *in vivo* conditions	Monolayers are composed mainly of living cells; since dead cells are detached and easily removed from culture
	Gene expression profiles are more comparable to *in vivo* environment	Often proliferate at a faster rate
	Cell morphology is closely similar to its natural shape	More cells are likely to be in the same stage of cell cycle
	Flexible: culture conditions can be modified to recapitulate a particular microenvironment	Well-established
	More stable in culture and can survive longer, which is suitable for long-term studies	Easy to observe, measure and analyze
	Cost effective; based on the assay	Cheap
	Bridges the gap between *in vitro* assays and *in vivo* studies	
	Minimize the use of animal models	
	Suitable for high-throughput platforms	
Demerits	Cells are existing in various cell cycle stages; including proliferation, apoptosis, and necrosis	Abnormal morphology of cells (flat and stretched) compared to *in vivo*
	Cells (especially those in the core) do not receive equal amounts of nutrients, oxygen, or growth factors due to the lack the complex vascular systems	Cells do not mimic the physiological *in vivo* microenvironment or organ-specific structural organizations
	Risk of transmission of infections agents from living-derived materials used to fabricate scaffolds	Display different gene profiles compared to *in vivo* environment
	Reproducibility is an issue due to batch-to-batch variations of biomimetic scaffolds	Survive for short time before trypsinization
	Microscopy imaging quality is a challenge based on scaffold sizes and material transparency	
	Optimization of different protocols	
	Expensive for large-scale studies and high throughput assays	

**Table 2:** Features of microfluidic systems.

**Merits**	**Demerits**
Mimic *in vivo* microenvironment, including dynamic conditions and shear forces	Need well-experience personnel
Tailoring the needs of single-cell or multi-cellular cultures in the same chip	Several optimizations
Reduce contamination risk	Microfabrication is a challenge
High throughput experimentations	
Controlled co-culture conditions via costumed chip architectures	
Direct coupling to downstream analysis systems	
Real-time	
Single cell handling flexibility	
Feasibility to track cell-cell interaction, cell proliferation, progress of infection and virus spread using live-cell imaging	
Very cost effective: it utilizes reagents in nanoscale volumes	
Enable better cell growth and proliferation in 3D culture systems	
Incorporate analytical biosensors into the culture platform	

## Author Contributions

MK wrote part of the introduction and the contact and flow chamber assay sections. SP wrote part of the abstract and introduction, nasal explant and EREC sections. KO edited and corrected the manuscript. WA designed the article, edited, wrote and corrected the manuscript.

### Conflict of Interest Statement

The authors declare that the research was conducted in the absence of any commercial or financial relationships that could be construed as a potential conflict of interest.
